# The value of repeat neuroimaging for epilepsy at a tertiary referral centre: 16 years of experience^☆^

**DOI:** 10.1016/j.eplepsyres.2013.02.022

**Published:** 2013-08

**Authors:** Gavin P. Winston, Caroline Micallef, Brian E. Kendell, Philippa A. Bartlett, Elaine J. Williams, Jane L. Burdett, John S. Duncan

**Affiliations:** a Epilepsy Society MRI Unit, Department of Clinical and Experimental Epilepsy, UCL Institute of Neurology, London, United Kingdom; b Lysholm Department of Neuroradiology, National Hospital for Neurology and Neurosurgery, London, United Kingdom

**Keywords:** Structural MRI, Focal cortical dysplasia, Refractory epilepsy, Epilepsy surgery

## Abstract

**Purpose:**

Magnetic resonance imaging (MRI) is the investigation of choice for detecting structural lesions that underlie and may accompany epilepsy. Despite advances in imaging technology, 20–30% of patients with refractory focal epilepsy have normal MRI scans. We evaluated the role of repeated imaging with improved MRI technology – an increase in field strength from 1.5 T to 3 T and superior head coils – in detecting pathology not previously seen.

**Methods:**

Retrospective review of a large cohort of patients attending a tertiary epilepsy referral centre who underwent MRI at 1.5 T (1995–2004) and subsequently 3 T (2004–2011) with improved head coils. Scan reports were reviewed for the diagnoses and medical notes for the epilepsy classification.

**Results:**

804 patients underwent imaging on both scanners, the majority with focal epilepsy (87%). On repeat scanning at 3 T, 37% of scans were normal and 20% showed incidental findings. Positive findings included hippocampal sclerosis (13%), malformations of cortical development (8%), other abnormalities (4%) and previous surgery (18%). A total of 37 (5%) relevant new diagnoses were made on the 3 T scans not previously seen at 1.5 T. The most common new findings were hippocampal sclerosis, focal cortical dysplasia and dysembryoplastic neuroepithelial tumour. These findings affected patient management with several patients undergoing neurosurgery.

**Conclusions:**

The higher field strength and improved head coils were associated with a clinically relevant increased diagnostic yield from MRI. This highlights the importance of technological advances and suggests that rescanning patients with focal epilepsy and previously negative scans is clinically beneficial.

## Highlights

• 20–30% of patients with refractory focal epilepsy have normal MRI scans.
• We evaluated the role of repeated MRI with better technology in detecting pathology.
• 804 patients underwent MRI at 1.5T and subsequently at 3T with superior head coils.
• Relevant new diagnoses were made in 37 (5%) and affected patient management.
• Rescanning patients with focal epilepsy and previously normal MRI is beneficial.

## Introduction

Epilepsy has a prevalence of approximately 1% (Sander and Shorvon, 1996). Over half have focal epilepsy and around one-third are refractory to medical treatment (Semah and Ryvlin, 2005). Magnetic resonance imaging (MRI) is the imaging modality of choice to detect possible structural lesions underlying the epilepsy, to assess comorbidities and to evaluate individuals with medically refractory epilepsy for potential surgery. UK guidelines recommend MRI in all patients with epilepsy, except those children and young persons with idiopathic generalised epilepsy who respond to drug treatment (National Institute for Health and Clinical Excellence, 2012).

Identification of a lesion is crucial for the consideration of surgical treatment and is associated with a greater chance of seizure freedom following surgery (Kuzniecky et al., 1993, Mosewich et al., 2000). In hippocampal sclerosis, co-existent abnormalities (“dual pathology”) are common, affecting 15% of adults and up to 67% of children (Bocti et al., 2003). Correct recognition of abnormalities influences management and prognosis.

Despite advances in imaging technology, around 20–30% of patients with refractory focal epilepsy have normal MRI scans (“MRI-negative”) (Duncan, 2010). It is widely held that an increase in field strength from 1.5 T to 3 T is associated with greater sensitivity for the detection of lesions. However, comparisons of imaging in patients with epilepsy at different field strengths are limited to small series of patients being considered for surgery and are not unanimous in their conclusions. In a series of 40 patients undergoing surgical evaluation, 3 T MRI identified a lesion in 65% of the patients considered MRI negative at 1.5 T (Knake et al., 2005). In contrast another study of 37 patients undergoing surgical evaluation failed to demonstrate any superiority of 3 T MRI over 1.5 T MRI (Zijlmans et al., 2009). Other technological developments, in particular changes in the design of head coils and gradients, may contribute but have not been assessed in patients with epilepsy.

The present study presents a retrospective review of a large cohort of patients attending a tertiary epilepsy referral centre who had undergone MRI at 1.5 T and later at 3 T with improved head coils. The aims were to determine the range of underlying pathologies and to identify that proportion in whom modern scanner technology yielded additional findings and how these affected clinical management.

## Methods

The National Hospital for Neurology and Neurosurgery in conjunction with the Epilepsy Society at Chalfont runs a large tertiary referral epilepsy service with 1300 new referrals for epilepsy and 50 undergoing surgery per year. The Epilepsy Society MRI Unit opened in 1995 with a 1.5 T General Electric Horizon Echospeed scanner (GE Medical Systems, Milwaukee, USA) employing quadrature coils. This was replaced by a 3 T General Electric Signa Excite HDx scanner (GE Medical Systems, Milwaukee, USA) using an 8-channel phased array receive coil in 2004. A dedicated epilepsy protocol MRI acquisition was used throughout (Supplementary Tables 1 and 2). During the period 1995–2010, two neuroradiologists reported the scans and since November 2010, a single neuroradiologist has reported the scans.

The MRI database was queried for the periods May 1995–June 2004 (16,692 bookings for the 1.5 T scanner) and July 2004–July 2011 (8823 bookings for the 3 T scanner) to identify patients who had undergone cranial MRI on the 1.5 T scanner and subsequently on the 3 T scanner. Scan reports were reviewed to determine the diagnosis and in the case of multiple scans on one scanner the most recent diagnosis was used. Only patients in whom the indication for scanning was epilepsy and with reports available on both scanners were included. The medical notes were reviewed to determine the treating clinician's classification of the epilepsy (focal, generalised or unclassified). Printed films of the 1.5 T scans were reviewed and the most recent 3 T scans were reviewed electronically to identify illustrative cases. The 1.5 T electronic data are no longer available and the printed films represent only a subset of the sequences acquired.

This retrospective evaluation of clinically acquired data was considered by the Joint Research Ethics Committee of the National Hospital for Neurology and Neurosurgery and Institute of Neurology to be audit/service evaluation so individual patient consent was not required.

## Results

804 patients who underwent imaging on both scanners were identified. The majority suffered focal epilepsy (697, 87%), with the remainder either generalised (66, 8%) or unclassified epilepsies (41, 5%).

37% of the mostly recently performed 3 T scans were normal (37%) and 20% had only incidental findings (Table 1). All patients with generalised epilepsy were in this group. The remainder showed abnormalities (13% hippocampal sclerosis with or without other pathology, 8% malformations of cortical development, 4% other) or previous surgery (18%, predominantly temporal).

**Table 1:** Diagnosis on most recent 3 T scan.

Diagnosis	Number (percentage)
**Normal**	**299 (37%)**
**Hippocampal sclerosis**	**101 (13%)**
Unilateral	50
Unilateral with dual pathology	37
Bilateral	10
Bilateral with dual pathology	4
**Malformations of cortical development**	**61 (8%)**
Tuberous sclerosis	7
Focal cortical dysplasia	22
Dysembryoplastic neuroepithelial tumour	18
Heterotopia	5
Polymicrogyria	3
Unspecified	6
**Other abnormality**	**36 (4%)**
Cavernoma	9
Glioma	7
Meningioma (causative)	1
Hypothalamic hamartoma	3
Haemorrhage	2
Hydrocephalus	1
Unspecified lesion	13
**Non-specific/incidental**	**162 (20%)**
Cerebral/cerebellar atrophy	47
Damage (unspecified, predominantly traumatic)	47
Infarct/ischaemic damage	32
White matter lesions	28
Meningioma (incidental)	3
Other incidental	5
**Post-surgical**	**145 (18%)**
Frontal	20
Temporal	119
Parietal	4
Occipital	2

**Figure 1:**
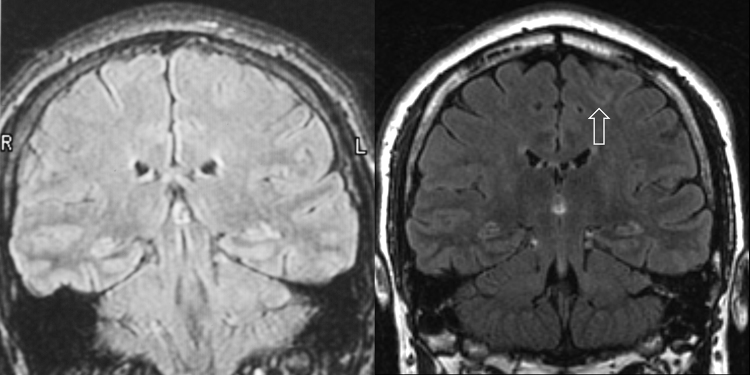
Refractory frontal lobe epilepsy due to left superior frontal sulcus focal cortical dysplasia. Patient with refractory frontal lobe epilepsy from the age of 12 years. Imaging at 1.5 T at the age of 20 in 2002 was reported as normal (A), but EEG and ictal SPECT were suggestive of a left frontal lobe onset. Subsequent 3 T imaging in 2009 revealed focal cortical dysplasia of the left superior frontal sulcus (B) despite the movement artefact. Images are coronal FLAIR images at the level of the posterior thalami. Resective surgery resulted in seizure freedom with histology confirming focal cortical dysplasia type IIb.

**Figure 2:**
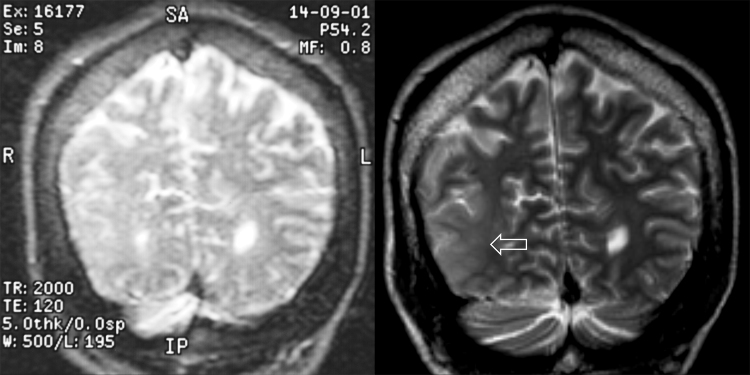
Refractory partial epilepsy with right inferior parietal malformation of cortical development. Patient with refractory partial epilepsy and learning disability. Imaging in 2001 at 1.5 T was reported as normal (A) but subsequent imaging in 2004 at 3 T revealed an extensive right inferior parietal malformation of cortical development (B). Images are coronal T2-weighted images at the posterior edge of the occipital horns.

**Figure 3:**
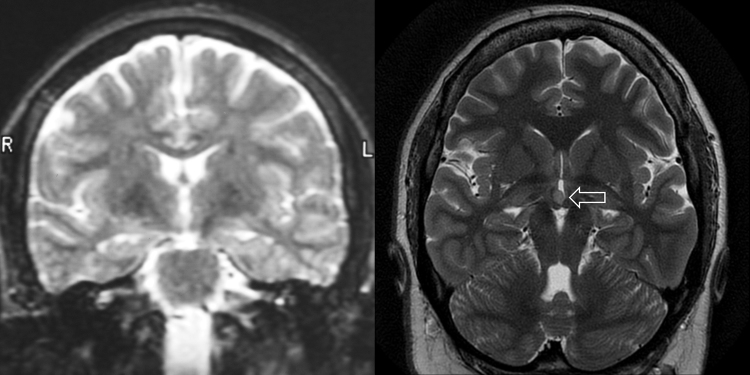
Complex motor seizures due to a hypothalamic hamartoma. Patient with complex motor seizures from the age of 2 years. Imaging at 1.5 T at the age of 22 years in 2001 was reported as normal (A) with the hypothalamic hamartoma only being identified on 3 T imaging in 2011 (B). Image A is coronal T2-weighted images at the level of mammillary bodies and temporal stems and image B is a coronal oblique PROPELLER (periodically rotated overlapping parallel lines with enhanced reconstruction) sequence. Gelastic seizures were identified in hindsight and the patient has undergone gamma knife surgery.

A total of 97 (12%) new diagnoses were found with the 3 T scans that were not made on the previous 1.5 T scans (Table 2). Whilst many were incidental, they were of direct relevance in 37 patients (5%, Figure 1, Figure 2, Figure 3), particularly hippocampal sclerosis, focal cortical dysplasia and dysembryoplastic neuroepithelial tumour (DNET).

**Table 2:** New diagnoses made between 1.5 T and 3 T scans, and surgical decision.

Diagnosis	Number (percentage)	Surgical decision
**Hippocampal sclerosis**	**13 (1.6%)**	
Unilateral hippocampal sclerosis (HS)	10	ATLR (2)
Bilateral HS (previously unilateral HS)	3	
**Malformations of cortical development**	**19 (2.4%)**	
Tuberous sclerosis	1	Tuber resection (1)
Focal cortical dysplasia	13	Surgery (3), ICR – unsuitable (1), declined ICR (2), pursuing AED (2), considered unsuitable (1), undergoing further investigation (4)
Dysembryoplastic neuroepithelial tumour	4	ICR – unsuitable (1), declined surgery (2), pursuing AED (1)
Unspecified	1	Unsuitable (1)
**Other abnormality**	**5 (0.6%)**	
Meningioma (recurrence)	1	Seizure free (1)
Hypothalamic hamartoma	1	Gamma knife surgery (1)
Haemorrhage (significant)	1	Declined surgery (1)
Hydrocephalus	1	Insertion of shunt (1)
Unspecified lesion	1	ICR – unsuitable (1)
**Non-specific/incidental**	**60 (7.5%)**	
Cerebral/cerebellar atrophy	22	
Traumatic damage	14	
Infarct/ischaemic damage	9	
White matter lesions	3	
Meningioma (incidental)	5	
Haemorrhage (incidental)	4	
Other incidental	3	

Abbreviations: AED, antiepileptic drugs; ATLR, anterior temporal lobe resection; ICR, intracranial EEG recording.

These new findings did affect patient management. Several patients underwent surgical procedures, including anterior temporal lobe resection, removal of an epileptogenic tuber, gamma knife surgery for hypothalamic hamartoma and insertion of a shunt for hydrocephalus (Table 2). Of the 13 patients newly diagnosed with focal cortical dysplasia, 3 underwent surgery, 1 underwent intracranial recording but was unsuitable for resection, 2 declined intracranial recording, 2 are pursuing anti-epileptic drugs currently, 1 was considered unsuitable and 4 are currently undergoing further presurgical investigations.

Progression of the underlying abnormality (e.g. atrophy, ischaemia) was observed in a further 9 patients. A change in diagnosis was made in 18 patients and 91 patients (11%) had undergone surgery, predominantly temporal (79 patients). The changes in diagnosis were mostly reinterpretation of the imaging data (12 patients), for example from DNET to glioma. These changes were histologically confirmed in the two patients proceeding to surgery. In the remaining 6 patients a previously reported abnormality was subsequently considered normal (hippocampal sclerosis in 5 cases). Overall the reports showed a change in 206 patients (26%).

## Discussion

### Key findings

Magnetic resonance imaging plays a key role in the assessment of patients with epilepsy and in this study a relevant finding was detected in 30% of patients who had not already undergone surgery. This exceeds a previously reported yield for relevant abnormalities of 18% at 3 T MRI in focal epilepsy (Craven et al., 2011), although referral and selection bias in the population scanned will affect the yield.

Significant improvements in imaging technology have occurred over recent years and a number of factors may influence the diagnostic yield. The key finding in this study was that 3 T scans with modern head coils and gradients yielded a relevant new diagnosis in 5% of those patients who had previous 1.5 T scans in the same centre and which had been reported as normal, and a further, smaller proportion had diagnoses clarified that were not due to disease progression between the two scans.

### Factors affecting the diagnostic yield

High resolution fast sequences at 1.5 T, including 3D volumetric T1-weighted and T2-weighted fast spin echo (FSE) sequences (Barkovich et al., 1995) and fluid-attenuated inversion recovery (FLAIR) imaging improves the detection of relevant pathology (Bergin et al., 1995) so it is important to implement a specialist epilepsy imaging protocol (Recommendations for neuroimaging of patients with epilepsy, 1997, Guidelines for neuroimaging, 1998). This detects pathology in 85–94% (McBride et al., 1998, Von Oertzen et al., 2002) of patients with focal epilepsy who were MRI-negative with conventional sequences at 1.5 T.

Two major technical developments are the increase in field strength from 1.5 T to 3 T (Alvarez-Linera, 2007) and the change in coil design from quadrature to phased array coils. The increased signal to noise ratio (SNR) from the latter can increase diagnostic yield at 1.5 T (Grant et al., 1998, Goyal et al., 2004) and in combination with doubling the field strength is estimated to increase SNR by a factor of 6–8 (Knake et al., 2005). This large increase in SNR allows faster imaging for a given resolution, a higher resolution for a given scan time or a combination of the two (Alvarez-Linera, 2007). Of particular importance is the ability to acquire thinner slices and our protocol has recently been extended by the addition of 3.5 mm thick axial FLAIR and 3 mm thick coronal FSE T2 imaging.

Diagnostic yield can be further improved by scans being read by expert neuroradiologists working in concert with neurologists with expertise in epilepsy. Detection of abnormalities on conventional 1.5 T MRI images had a sensitivity of 39% with non-experts compared to 50% in experts (Von Oertzen et al., 2002). This increased further to 91% when experts were shown high resolution studies. However, some abnormalities escape visual detection even by experienced observers and the introduction of computer-based image analysis techniques such as voxel-based FLAIR (Focke et al., 2008) or surface-based morphological analysis for focal cortical dysplasia (Besson et al., 2008, Thesen et al., 2011) may further increase the yield.

### Focal cortical dysplasia

The increased detection of hippocampal sclerosis at 3 T is likely a combination of improved sensitivity and the development over time of this pathology between the scans, which can be further studied by T2 relaxometry and hippocampal volumetry (Woermann et al., 1998). However, malformations of cortical development are likely to have been present throughout and the increased detection of focal cortical dysplasia (FCD) represents a significant advance which deserves special mention. The correct identification of FCD is challenging. In a retrospective series of pathologically proven FCD, the MRI was reported as normal in 34% (Tassi et al., 2002). Standard imaging fails to identify FCD in up to 80% of patients when the abnormality lies in the depth of a sulcus (Besson et al., 2008). The improved SNR at 3 T improves the detection of focal abnormalities such as FCD through better definition of the grey/white matter junction (which becomes blurred in FCD) and a more uniform signal intensity in normal cortex (in contrast to hyperintense signal within the dysplastic region).

### Previous studies

Two studies have compared 1.5 T and 3 T scans in patients with epilepsy and reached contradictory conclusions. In the first a series of 40 patients undergoing presurgical evaluation underwent scans on a Siemens 3 T scanner at a specialist centre with phased array coils (Knake et al., 2005). In comparison to prior standard 1.5 T MRI performed and reported elsewhere, lesions were detected in 65% of previously MRI negative patients and clinical management was affected in 38%. However, four variables were different – field strength, availability of a phased array coil, use of a specialist epilepsy protocol and reporting by an experienced observer. Given these numerous differences and the inclusion of only patients being considered for surgery, it is not surprising that there was a higher yield of new diagnoses in previously MRI-negative patients.

The second study included 37 patients being considered for surgery who were scanned on 1.5 T and 3 T scanners both using phased array coils and reported by the same observers (Zijlmans et al., 2009). Thus there were fewer technical differences between the scans. This in conjunction with the selection of only patients considered ineligible for surgery following 1.5 T scans might explain the failure to demonstrate increased yield at 3 T. Whilst dysplasia was detected more frequently, hippocampal sclerosis was reported less often.

### Strengths and limitations

In the context of limited and contradictory previous literature, the present study represents the largest comparison of repeat imaging in patients with epilepsy at a tertiary referral centre over a period of 16 years. It is not possible to disentangle the relative contributions to diagnostic yield of an increase in field strength from the introduction of phased array coils (used on the 3 T scanner, but not the 1.5 T scanner). However, it is not feasible to apply a prospective study to such a large cohort with the previous studies only assessing 37 (Zijlmans et al., 2009) or 40 patients (Knake et al., 2005).

The particular strengths of the current study are the size of the cohort, the use of dedicated epilepsy protocols throughout and the scan reporting by experienced neuroradiologists, with the majority of scans at both field strengths being reported by the same two observers.

In the current study, the scans were clinically reported with knowledge of the clinical details, rather than blind, and only the scan reports rather than the imaging itself were reviewed for this study. In some cases it may be possible in hindsight to identify a lesion only identified with the 3 T scan on the earlier 1.5 T scan. However, this does not represent clinical practice and the full 1.5 T imaging data from the 1990s are not available for review. This chance is minimised by the use of the same two experienced neuroradiologists for the majority of the reporting period and many of the new diagnoses obtained were those that theoretically benefit from the improved scan technology. Finally, some selection bias may have been introduced as those having repeated scans may be more likely to have been previously MRI negative and to have epilepsy that had not gone into remission, and thus be biased to those with a higher likelihood of structural pathology than those who remitted and who would thus be less likely to be rescanned.

## Conclusions

In conclusion, this is the largest study of a cohort of patients with epilepsy undergoing MRI at 1.5 T and subsequently at 3 T with improved head coils with both employing a specialist epilepsy protocol at a tertiary referral centre. 3 T MRI identified a relevant abnormality in around 30% patients who had not already undergone surgery. In 5% of patients, a relevant diagnosis was reached with the 3 T scan which had not been reported on the previous 1.5 T scan. The new information contributed to clinical management, particularly in the detection of focal cortical dysplasia.

The yield of diagnostic imaging is dependent upon many factors, including high resolution sequences, magnetic field strength, phased array coils, gradient performance, a specialist epilepsy protocol and dedicated neuroradiologists and may be increased further by the introduction of computer-based analysis techniques. The point of practical importance is to rescan individuals with previously unclear MRI using optimal technology.

## Conflicts of interest

John Duncan has served as a consultant for GE Healthcare on the development of PET tracers. The remaining authors have no conflict of interest.
