# Supplementary material for: The value of repeat neuroimaging for epilepsy at a tertiary referral centre: 16 years of experience
Source: Epilepsy Res. 2013 Aug;105(3):349–55. doi: 10.1016/j.eplepsyres.2013.02.022 (PMC3888924; doi:10.1016/j.eplepsyres.2013.02.022)
Supplement: Supplementary file 1 [file mmc1.docx]

**Supplementary Table 1**

**Epilepsy protocol on 1.5T GE Horizon Echospeed scanner**

| **Sequence** | **Plane** | **Acquisition time (mm:ss)** | **Slice thickness / spacing (mm)** | **Number of slices** | **Matrix size** | **Field of view (cm)** |
| --- | --- | --- | --- | --- | --- | --- |
| T1 FLAIR | Sagittal | 2:47 | 5 / 2 | 17 | 256x256 | 24x24 |
| FSPGR 3D | Coronal oblique | 6:56 | 1.5 / 0 | 124 | 192x256 | 18x24 |
| T2 FLAIR | Coronal oblique | 8:04 | 5 / 0 | 28 | 192x256 | 18x24 |
| PD/T2 SE | Coronal oblique | 10:24 | 5 / 0 | 28 | 192x256 | 18x24 |
| High resolution FSPGR (temporal lobe only) | Coronal oblique | 8:45 | 1.5 / 0 | 60 | 384x512 | 18x24 |

Key: FLAIR = fluid-attenuated inversion recovery, FSPGR = fast spoiled gradient recalled, PD = proton density, SE = spin echo

**Supplementary Table 2**

**Epilepsy protocol on 3T GE Signa Excite HDx scanner**

| **Sequence** | **Plane** | **Acquisition time (mm:ss)** | **Slice thickness / spacing (mm)** | **Number of slices** | **Matrix size** | **Field of view (cm)** |
| --- | --- | --- | --- | --- | --- | --- |
| T1 FLAIR | Sagittal oblique | 1:28 | 5 / 2 | 17 | 320x224 | 24x24 |
| FSPGR 3D | Coronal oblique | 7:30 | 1.1 / 0 | 170 | 256x256 | 18x24 |
| T2 FLAIR | Coronal oblique | 2:56 | 5 / 0 | 32 | 224x256 | 18x24 |
| PD/T2 FRFSE | Coronal oblique | 2:01 | 5 / 0 | 32 | 256x256 | 18x24 |
| FGRE T2* | Coronal oblique | 2:12 | 5 / 0 | 32 | 192x192 | 18x24 |
| FSE T2 | Axial oblique | 1:23 | 5 / 0 | 30 | 256x512 | 18x24 |
| PROPELLER (selected patients only) | Coronal oblique | 3:20 | 2 / 0 | 32 | 416x416 | 22x22 |

Key: FGRE = fast gradient echo, FLAIR = fluid-attenuated inversion recovery, FRFSE = fast recovery fast spin echo, FSE = fast spin echo, FSPGR = fast spoiled gradient recalled, PD = proton density, PROPELLER = periodically rotated overlapping parallel lines with enhanced reconstruction, SE = spin echo
